# Improving Care for Individuals Living With Intellectual Developmental Disabilities and Dementia

**DOI:** 10.1093/geroni/igab046.1982

**Published:** 2021-12-17

**Authors:** Donna Barrett

**Affiliations:** Summit County Public Health, Akron, Ohio, United States

## Abstract

Ultimately, transformation of communities can only occur through educational efforts delivered to specific community sectors. Although the portion of people with Intellectual Developmental Disabilities who develop dementia as they age is equal to that of the general population, individuals with Down syndrome are at a much higher risk. This symposium will describe how a county health department partnered with the local County Board of Developmental Disabilities to systematically incorporate Dementia Friends for Intellectual Developmental Disabilities with Alzheimer’s disease training to their staff and provider network. We will describe who to get on board with the idea, how to organize, and how to deliver specific trainings. Outcomes related to increase in participant knowledge, increases in service provision and outcomes related to staff mentoring will be discussed.

